# Characterization of Proanthocyanidins in Stems of *Polygonum multiflorum* Thunb as Strong Starch Hydrolase Inhibitors

**DOI:** 10.3390/molecules18022255

**Published:** 2013-02-18

**Authors:** Hongyu Wang, Lixia Song, Shengbao Feng, Yuancai Liu, Gang Zuo, Fuli Lai, Guangyuan He, Mingjie Chen, Dejian Huang

**Affiliations:** 1 Food Science and Technology Program, Department of Chemistry, National University of Singapore, 3 Science Drive 3, Singapore, 117543, Singapore; 2 The Genetic Engineering International Cooperation Base of Chinese Ministry of Science and Technology, Chinese National Center of Plant Gene Research (Wuhan) HUST Part, College of Life Science and Technology, Huazhong University of Science & Technology, Luoyu Road 1037, Wuhan 430074, Hubei, China; 3 Jing Brand Company, 169 Daye Avenue, Daye 435100, Hubei, China

**Keywords:** *P. multiflorum*, proanthocyanidins, α-amylase, (epi)catechin

## Abstract

Characterzation of polyphenolic compounds in the stems of *P. multiflorum* was conducted using HPLC, high resolution LC-MS and LC-MS^n^. Proanthocyanidins in particular were isolated in 4.8% yield using solvent extraction followed by Sephadex LH-20 fractionation. HPLC analysis using a diol column revealed oligomers (from dimer to nonamer) as minor components, with (epi)catechin monomeric units predominating, and oligomers with higher degree of polymerization being dominant. Thiolysis treatment of the proanthocyanidins using mercaptoacetic acid produced thioether derivatives of (epi)catechin as the major product and a mean value of the degree of polymerization of 32.6 was estimated from the ratio of terminal and extension units of the (epi)catechin. The isolated proanthocyanidins were shown to strongly inhibit α-amylase with an acarbose equivalence (AE) value of 1,954.7 µmol AE/g and inhibit α-glucosidase with an AE value of 211.1 µmol AE/g.

## 1. Introduction

The root of Foti or *Polygonum multiflorum* Thunb has found broad use as a tonic and herb in China for many traditional uses, including treatment of hair loss [1], replenishing the vital essence of blood, curing malaria, and clearing away toxins [2]. The main active constituents in the roots have been characterized as polyphenolic compounds, including hydroxyanthraquinones, stilbenoids and proanthocyanidins [3,4], which are responsible for the radical scavenging activity of the roots [5]. The mice fed with root extracts of *Polygonum multiflorum* Thunb were shown to possess improved learning and memory ability and reduced oxidative stress status as measured by malondialdehyde concentrations [6]. Consistent with this work, it was found that antioxidants in *Polygonum multiflorum* Thunb roots were mostly extractable using water [7], indicating the active components are hydrophilic compounds including 2,3,5,4'-tetrahydroxystilbene 2-*O*-*β*-d-glucopyranoside, which is the main antioxidant in the root [5]. In addition to the radical scavenging activity, Sang and workers have shown that stilbene glucoside from *Polygonum multiflorum* Thunb can trap methylglyoxal, the toxic reactive carbonyl species that can react with proteins and lead to the formation of advanced glycation endproducts (AGES) [8]. Since methylglyoxal toxicity is a major cause of diabetic complications [9], mitigation of its harmful effect through a herbal medicine would be a potential useful application of *Polygonum multiflorum* Thunb extracts.

The existing literature on bioactive components strongly substantiates the health promotion effects of the roots of *Polygonum multiflorum* Thunb. In addition to the roots, the stems, or Fleeceflower Stem tuber of the plant have been used as traditional herb for over a thousand years for health promotion purposes. In contrast to the roots, much less research has been carried out on characterization of bioactive components in the stems. Although it has been reported that the stem of *Polygonum multiflorum* Thunb, exhibited even more potent antioxidant capacity than the root of *Polygonum multiflorum* Thunb [10], its polyphenolic profile is not fully characterized. Reported herein are the results of our attempts to elucidate the polyphenolic compound profiles in the stems of *Polygonum multiflorum* Thunb. In particular, using a high throughput assay as a guide, we identified and characterized the proanthocyanidins in the stems as potent starch hydrolase inhibitors, which has potential as functional ingredients in reducing postprandial hyperglycemia.

## 2. Results and Discussion

### 2.1. HPLC and High Resolution Mass Spectra Characterization of Polyphenolic Compounds Extract

**Figure 1:**
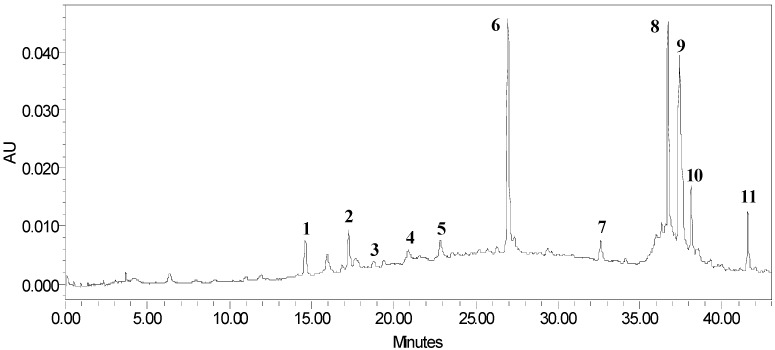
HPLC chromatogram of phenolic compounds in *P. multiflorum* Thunb.

The HPLC chromatogram of a methanolic extract of *P. multiflorum* Thunb is shown in Figure 1. Eleven peaks were characterized based on the HR-LC-MS results and MS^n^ fragmentation behaviors (Table 1). Three classes of compounds were identified.

#### 2.1.1. Flavan-3-ols

Compounds in peaks **1**, **2**, **3** and **5** were tentatively assigned as proanthocyanidins according to the accurate [M−H]^−^ anion mass and the fragmentation patterns of the ESI-MS^n^ (anion mode). Compounds **1**, **2** and **5** have accurate *m/z* [M−H]^−^ values of 577.1366, 865.2010 and 1153.2572, respectively, and were tentatively assigned as dimeric, trimeric and tetrameric proanthocyanidins. The compound in peak **3** has an accurate *m/z* [M−H]^−^ of 1017.2071 and was tentatively assigned as a proanthocyanidin trimer with two (epi)catechin units and one (epi)catechin gallate unit.

**Table 1:** LC-HR-MS and LC-MS^n^ data for compounds.

Peak No.	Compound identity	*Measured HRMS m/z* [M−H]^−^	Predicted *HRMS m/z* [M−H]^−^	Mass Error (ppm)	MS^n^ fragmentations [M−H]^−^
1	Dimer, (epi)C_2_	577.1366	577.1351	−2.5	451,425,287
	Molecular formula	C_30_H_26_O_12_			
2	Trimer, (epi)C_3_	865.2010	865.1985	−2.8	575,407
	Molecular formula	C_45_H_38_O_18_			
3	Trimer, (epi)C_2_+(epi)CG	1017.2071	1017.2095	2.4	847,729, 677,577,287
	Molecular formula	C_52_H_42_O_22_			
4	Ester of phenylpropanoid and phenylethanoid glucoside	839.2396	839.2404	1.0	677,641,515
	Molecular formula	C_41_H_43_O_19_			
5	Tetramer, (epi)C_4_	1153.2572	1153.2619	4.1	863,575,407
	Molecular formula	C_60_H_50_O_24_			
6	Tetrahydroxystilbene glucoside	405.1190	405.1191	0.3	243,137
	Molecular formula	C_20_H_22_O_9_			
7	Methyl-*O*-digalloyl-glucoside	497.0934	497.0937	0.6	345,327
	Molecular formula	C_21_H_22_O_14_			
8	Emodin-*O*-glucoside	431.0982	431.0984	0.3	269,241
	Molecular formula	C_21_H_20_O_10_			
9	Physcion-*O*-glucoside	445.1149	445.1140	−1.9	283,268
	Molecular formula	C_22_H_22_O_10_			
10	Physcion	283.0599	283.0612	4.5	268,240
	Molecular formula	C_16_H_12_O_5_			
11	Emodin	269.0445	269.0455	3.9	241,224
	Molecular formula	C_15_H_10_O_5_			

The secondary mass of peak **1** gave three daughter ion peaks. A peak at *m/z* 451 arises from heterocyclic ring fission (HRF) of the heterocyclic rings; a peak at *m/z* 425 arises from retro-Diels-Alder fission (RDA-F) of the heterocyclic rings, and, lastly, a peak *m/z* 287 fragment is formed via quinone-methide (QM) cleavage of the interflavanic bond [11].

#### 2.1.2. Phenylpropanoids and Stilbenoids

**Figure 2:**
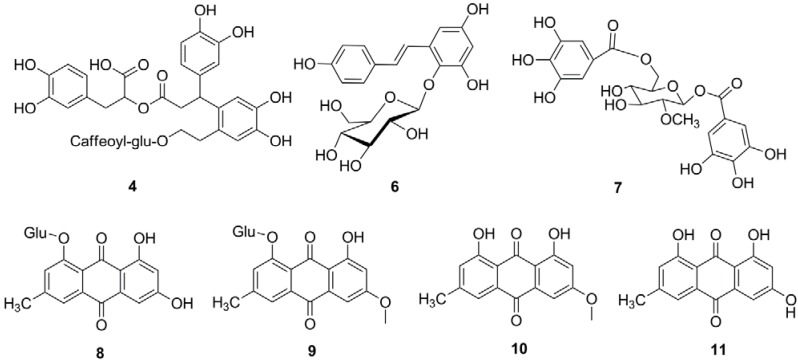
Structures of phenolic compounds identified in *P. multiflorum* Thunb. G, galloyl group; Glu, glucose.

Peak **4** has an accurate *m/z* [M−H]^−^ of 839.2396. Its corresponding formula is C_41_H_43_O_19_. The same compound was found in *Meehania urticifolia* [12]. The fragmentation by MS^2^ gave a peak at *m/z* [M−H]^−^ of 677 due to loss of one caffeoyl group from the molecular ion. The fragment ion at *m/z* [M−H]^−^ 515 might arise from the further loss of one glucose moiety. Therefore, peak **4** was tentatively assigned as the complex of a phenylpropanoid ester and phenylethanoid glucoside (Figure 2). The compound in peak **6** has the accurate *m/z* [M−H]^−^ of 405.1190. Its secondary ion at *m/z* 243 arises from the loss of one glucose moiety. Its secondary ion at *m/z* 137 might be derived from alkene bond cleavage, therefore, it was tentatively assigned as tetrahydroxystilbene glucoside. Based on this result, we can conclude that, the stems of the *Polygonum multiflorum* Thunb also contain significant amounts of the stilbene derivatives found in the roots.

#### 2.1.3. Anthraquinone Derivatives

Four compounds of this class were detected. Peaks **8** and **9** have accurate *m/z* [M−H]^−^ at 431.0982 and 445.1149. Their secondary ions are 269 and 283, respectively, which arises from the loss of one glucose moiety. Therefore, they were tentatively assigned as emodin glucoside and physcion glucoside. Peak **10** has an accurate *m/z* [M−H]^−^ of 283.0599. Its secondary ion at *m/z* 268 [M−H−15]^−^ was is suggested to be formed by elimination of a methyl group. The peak at *m/z* 240 [M−H−15−28]^−^ was suggested to be formed by further eliminating a CO group [13]. This is consistent with the reported fragmentation pattern of physcion [13]. The compound of peak **11** has an accurate *m/z* [M−H]^−^ of 269.0445. Its secondary ion at *m/z* 241 [M−H−28]^−^ arises from loss of a CO group. Its secondary ion at *m/z* 224 [M−H−28−17]^−^ arises from further loss of a hydroxyl group. Therefore, compound **11** was tentatively identified as emodin. The chemical structures were shown in Figure 2.

In addition to the three class of compounds, a hydrolysable tannin (peak **7**) was identified as methyl-*O*-digalloyl glucoside, with accurate [M−H]^−^ at *m/z* 497.0934. Its secondary ion at *m/z* 345 arises from the loss of one galloyl moiety. Its fragment ion at *m/z* 327 might be derived from further loss of water.

### 2.2. HPLC and LC-ESI-MS^n^ Characterization of Proanthocyanidins

**Figure 3:**
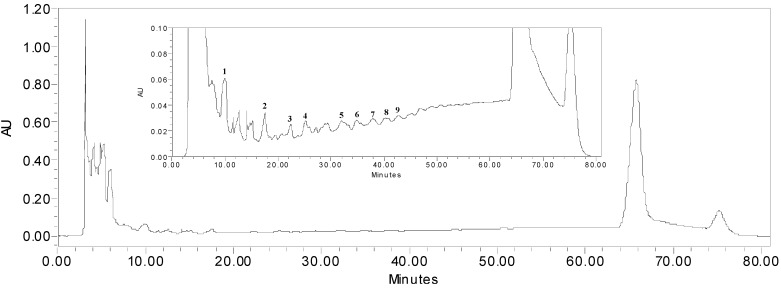
HPLC chromatogram of proanthocyanidins in *P. multiflorum* Thunb. The identities of the compounds are listed in Table 2.

Aqueous acetone extraction of *P. multiflorum* Thunb yielded crude proanthocyanidins. To fully characterize the compounds present in this extract, HPLC and LC-MS^n^ analysis were carried out using a diol column that was reported to be particularly effective in separating proanthocyanidins [11,12,13]. This worked nicely as shown by the resulting HPLC chromatogram (Figure 3).

We were able to assign the structures of the proanthocyanidins from peak **1** to **9** according to [M−H]^−^ anion mass and the fragmentation pattern of the ESI-MS^n^ (anionic mode). Compounds in peaks **1**, **2**, **4**, **6** and **8** have negative molecular ions [M−H]^−^ for dimer (*m/z* 577), trimer (865), tetramer (1153), pentamer (1441), and hexamer (1729) (epi)catechins with B type linkages, respectively. The type of linkage in proanthocyanidins can be identified readily by MS as A-type linkage (2 Da less) or B-type linkage. Peaks **3**, **5**, **7** and **9** have [M−H]^−^ of *m/z* 1017 (trimer), 1305 (tetramer), 1593 (pentamer) and 1881 (hexamer) derivatives of one gallate unit based on the molecular anion of 152 (galloyl) larger than the respective (epi)catechin oligomers (Table 2). The MS^2^ fragmentation patterns are in agreement with those of typical proanthocyanidins. Compared to the higher oligomer peaks at 66 min (Figure 3), these oligomer contents are insignificant. In addition, the amount of proanthocyanidins in the extract was estimated from HPLC chromatograms using epicatechin as a reference standard. The proanthocyanidins content was estimated to be 37 mg epicatechin equivalent per 100 mg extracts.

**Table 2:** Observed ions of proanthocyanidins in *P. multiflorum* Thunb by ESI-MS (anionic mode).

Peak number	Compound identity	*m/z* [M−H]^−^	MS^2^ fragmentation ions
1	Dimer, (epi)C_2_	577	451,425,287
2	Trimer, (epi)C_3_	865	575,407
3	Trimer, (epi)C_2_-(epi)CG	1017	847,729,577,287
4	Tetramer, (epi)C_4_	1153	863,575,407
5	Tetramer, (epi)C_3_-(epi)CG	1305	1179,1017,847,729,577,451
6	Pentamer, (epi)C_5_	1441	1315,1151,865
7	Pentamer, (epi)C_4_-(epi)CG	1593	1467,1441,1305,1017,727,575,447
8	Hexamer, (epi)C_6_	1729	1603,1577,1441,1151,1027,863.737,576
9	Hexamer, (epi)C_5_-(epi)CG	1881	1729,1593,1305,1177,1015,865,739

(epi)C, (epi)CG are the abbreviation for (epi)catechin, (epi)catechin gallate, respectively. The number in subscript indicates that the number of monomers.

### 2.3. Thiolysis of Proanthocyanidins for HPLC and LC-ESI-MS Analysis

**Figure 4:**
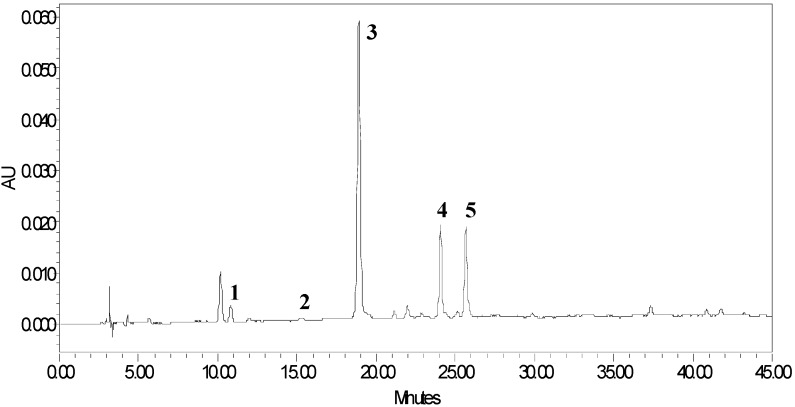
HPLC chromatographic profile of thiolysis products of proanthocyanidins. Catechin, **1**; epicatechin, **2**; catechin thioether, **3**; epicatechin thioether, **4**; (epi)catechin gallate thioether, **5**.

To determine the mean degree of polymerization, thiolysis reaction using mercaptoacetic acid as the nucleophilic agent were conducted to investigate the main components of the proanthocyanidins and the resulting HPLC profile of the thiolysis reaction products is given in Figure 4 [14].

The peaks were identified according to the retention times of the standard compounds and LC-ESI-MS results. The major product observed was the 4β-(carboxymethyl)sulphanyl-(−)-catechin methyl ester, followed by 4β-(carboxymethyl)sulphanyl-(−)-epicatechin methyl ester and 4β-(carboxymethyl)sulphanyl-(−)-(epi)catechin gallate methyl ester. These results showed that the extension units of proanthocyanidins in *P. multiflorum* Thunb consist of catechin, epicatechin and (epi)catechin gallate. For the terminal units, catechin and epicatechin were both observed from the thiolysis mixtures. By comparing the peak areas of the individual curves obtained from the HPLC, the mean DP of *P. multiflorum* Thunb proanthocyanidins was calculated to be 32.6 based on Equation (1):

(1) $$\text{Mean DP}=\frac{\left(\text{sum of extention units}+\text{sum of terminal units}\right)}{\left(\text{sum of terminal units}\right)}$$

It should be pointed out that, due to the different molar absorbance coefficient of thiolytic products 1–5, the actual mean degree of polymerization is different.

### 2.4. Starch Hydrolase Inhibition Activity

**Figure 5:**
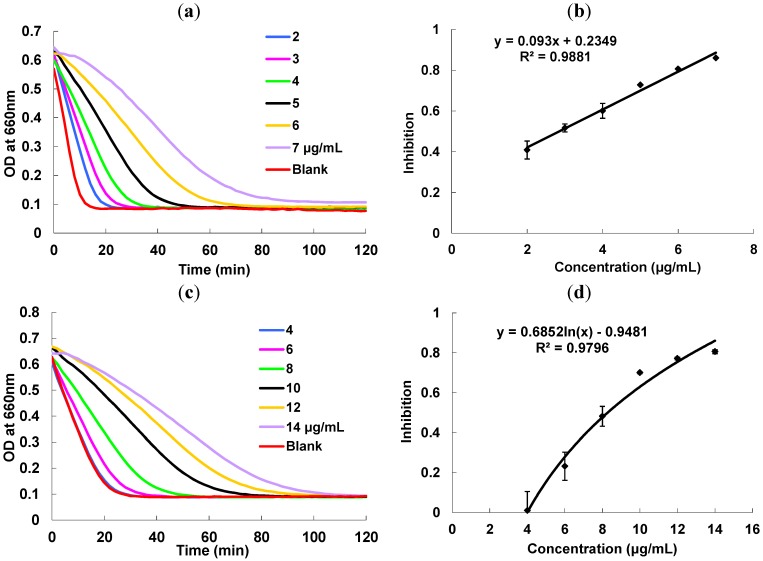
(**a**) Kinetic curves of α-amylase under different concentration ofproanthocyanidins extract; (**b**) α-amylase inhibition activity *vs.* concentrations of proanthocyanidins extract; (**c**) Kinetic curves of α-glucosidase under different concentration of proanthocyanidins extract; (**d**) α-glucosidase inhibition activity *vs.* concentrations of proanthocyanidins extract.

*P. multiflorum* proanthocyanidins extract showed strong inhibitory activity towards starch hydrolase as measured using a high throughput assay developed in our lab based on turbidity changes as a means to monitor starch hydrolysis kinetics [15]. The speed of the hydrolysis by α-amylase and α-glucosidase decreases with increasing proanthocyanidin concentrations (Figure 5). A linear dose-dependent correlation effect could be observed and the IC_50_ values were calculated as 2.9 and 7.4 µg/mL, respectively. By comparing with acarbose as a reference standard, the acarbose equivalent (AE) values of *P. multiflorum* proanthocyanidins are 1,954.7 ± 104.2 µmol AE/g for α-amylase (IC_50_ = 2.9 ± 0.15 µg/mL) and 211.1 ± 10.1 µmol AE/g for α-glucosidase (IC_50_ = 7.4 ± 0.35 µg/mL). Therefore, the proanthocyanidins are more selective towards α-amylase compared to acarbose. This warrants further study on how this selectivity would impact the side effects in human subjects in the future.

## 3. Experimental

### 3.1. Reagents and Instruments

α-Amylase (A3176, type VI-B, from porcine pancreas), corn starch (S4126), acarbose, α-glucosidase in the form of rat intestine acetone powder, mercaptoacetic acid were obtained from Sigma-Aldrich Chemical Co. (St. Louis, MO, USA). The stems of *Polygonum multiflorum* Thunb came from a cultivated product obtained in July 2011 from Jiuzhihe Township, Luotian County, Hubei, China. The voucher specimen was deposited in the Singapore Arboretum (number: SING-2011-553).

LC/MS spectra were acquired using a Bruker Amazon ion trap mass spectrometer (Billerica, MA, USA) equipped with a Dionex Ultimate 3000RS HPLC system (Bannockburn, IL, USA). The heated capillary and spray voltage were maintained at 250 °C and 4.5 kV, respectively. Nitrogen was operated at 80 psi for sheath gas flow rate and 20 psi for auxiliary gas flow rate. The full scan mass spectra from *m/z* 50–2,000 were acquired in negative ion mode with a scan speed of one second per scan. The MS^n^ collision gas was helium with collision energy of 30% of the 5V end cap maximum tickling voltage.

### 3.2. Extraction of Polyphenolic Compounds from P. multiflorum Thunb

The dried *P. multiflorum* Thunb (50 g) was meshed and extracted with 90% methanol (200 mL) for 4 h at room temperature. Filtration was performed to remove any insoluble solids. The supernatant was collected and the volatiles were evaporated by rotary evaporator and the dried residue was further dissolved in methanol for HPLC analysis.

### 3.3. Isolation of Proanthocyanidins

The proanthocyanidins were extracted based on an earlier report with a slight modification [16]. *P. multiflorum* Thunb (50 g) was dissolved in acetone-H_2_O (7:3, v/v) (3 × 500 mL), shaked for 4 h, and filtered to remove any insoluble solids. The filtrate was evaporated to dryness by rotary evaporation. The residue was dried to give 7.4 g of solid (14.8%). The solid were then purified on a LH-20 column. After the sample was loaded, the column was flushed with methanol-water (1:1, v/v) until the eluent turned colorless. The adsorbed proanthocyanidins were then eluted with acetone-H_2_O (7:3, v/v). The collected solution was evaporated to dryness to give 2.4 g (4.8%) of sample.

### 3.4. HPLC and Tandem Mass Spectrometry

The acetone-H_2_O (7:3) extract (10 mg) from *P. multiflorum* Thunb was dissolved in one mL methanol. Five μL of the solution was filtered through a 0.45 μm filter before injection into the LC/MS^n^ system equipped with a Develosil^®^ diol column (250 mm × 4.6 mm i.d., 5 μm, Seto, Aichi Japan). The elution conditions were as follows: flow rate, 1.0 mL/min; column temperature 35 °C; mobile phase A, 2% acetic acid in acetonitrile; mobile phase B, acidic aqueous methanol (CH_3_OH: H_2_O: HOAc, 95:3:2 v/v/v). The starting mobile phase condition is 7% B and held isocratic for 3 min. Subsequently, solvent B was ramped to 37.6% over 57 min and to 100% B 3 min thereafter. The condition was held at 100% B for seven min prior to returning to starting conditions (7% B) over six min. UV-VIS detector were set at 280 nm and epicatechin standard were run under the same condition for estimation of proanthocyanidins contents.

The 90% methanol extract (10 mg) from *P. multiflorum* Thunb was dissolved in one mL methanol. One µL of the solution was filtered through a 0.45 μm filter before injection into the LC/MS^n^ system equipped with a Atlantis^®^ T3 column (150 mm × 3.0 mm i.d., 3 μm, Ireland). The elution conditions were as follows: flow rate, 0.3 mL/min; column temperature 25 °C; mobile phase A, water (0.1% acetic acid); mobile phase B, acetonitrile. The starting mobile phase condition is 5% B; hold isocratic for 30 min. Subsequently, ramp solvent B to 80% over 5 min and to 100% B 5 min thereafter. Hold the conditions at 100% B for 5 min prior to returning to starting conditions (5% B) over 3 min. The wavelength of UV detector was set at 280 nm.

### 3.5. Thiolysis of the Proanthocyanidins for HPLC Analysis

This was performed following a reported method [13]. In a small glass vial, *P. multiflorum* Thunb proanthocyanidins (50 μL, 2 mg/mL in methanol) was mixed with methanol acidified with concentrated HCl (50 μL, 3.3%, v/v) and 100 μL of mercaptoacetic acid (5% v/v in methanol). The vial was sealed with an inert Teflon cap. The reaction was carried out at 40 °C for 30 min and then kept at room temperature for 10 h and kept in the freezer (−20 °C) until it was filtered through 0.45 μm filter and analyzed by HPLC. The thiolyzed proanthocyanidins were analyzed using LC/MS with a C18 column (250mm × 4.6mm i.d., 3 μm, Waters). The binary mobile phases consisted of A (2% acetic acid in water, v/v) and B (methanol), which were delivered in a linear gradient of B from 15 to 80% (v/v) in 45 min. The flow rate was set at 1.0 mL/min. The wavelength of UV detector was set at 280 nm.

### 3.6. α-Amylase and α-Glucosidase Inhibition Assay

The inhibition activities of *P. multiflorum* proanthocyanidins on α-amylase and α-glucosidase were monitored and quantified based on turbidity measurements according to previous work [15]. Inhibition assay solution consisted of enzyme solution (20 μL), inhibitor solution (20 μL), and 1% starch solution (60 μL). Acarbose was prepared in sodium phosphate buffer (0.1 M, pH 6.9). Proanthocyanidins were first dissolved in methanol and then diluted with sodium phosphate buffer to appropriate concentrations.

In a 96-well microplate, enzyme solution (20 μL) was preincubated with a series of concentrations of proanthocyanidin solution (20 μL) in a microplate reader for 15 min at 37 °C. The reaction was started by injecting starch solution (60 μL) using a 12-channel multichannel pipet. The turbidity change was immediately monitored at 660 nm for 2 h. The change of optical density (OD) was used as an indication of turbidity level. The percentage of inhibition was defined by Equation (2):

(2) $$\%\text{inhibition}=\frac{{\text{AUC}}_{\text{sample}}-{\text{AUC}}_{\text{control}}}{{\text{AUC}}_{\text{sample}}}\times 100$$

where AUC_sample_ is the area under the curve (AUC) of inhibitor; AUC_control_ is the area under the curve without inhibitors. The IC_50_ can be defined as the concentration of inhibitor that produces 50% inhibition of enzyme activity under the specified assay condition. It was obtained from interpolation of percentage of inhibition against inhibitor concentration curve.

### 3.7. Statistical Analysis

Statistical analysis was performed using Microsoft Excel. The data were expressed as mean of quintuple runs ± SD with replicate analysis.

## 4. Conclusions

We have shown that stems of *P*. *multiflorum* Thunb contain similar polyphenolic compounds to those found in roots, which are well studied and utilized as herbal medicine. It is remarkable that the stems contain high amounts of proanthocyanidins, predominantly composed of catechin and/or epicatechin units and with a very large mean degree of polymerization of 32.5. This is an ideal feature for converting the proanthocyanidins into value-added epicatechin derivatives [17]. The proanthocyanidins exhibit potent α-amylase and moderate α-glucosidase inhibitory activity. Stems of *P*. *multiflorum* Thunb are readily available and economical, and are under-utilized compared to the roots. Our results highlights the need for further research and application of polyphenolic compounds in the stems as starting materials for the preparation of epicatechin derivatives and as functional food ingredients.

## References

[1] China Pharmacopoeia Committee (2004). Pharmacopoeia of the People’s Republic of China.

[2] Ou M. (1996). Chinese English Dictionary of Traditional Chinese Medicine.

[3] Nanaka G.I., Miwa N., Nishioka I. (1982). Stilbene glycoside gallates and proanthocyanidins from *Polygonum multiflorum*. Phytochemistry.

[4] Yao S., Li Y., Kong L. (2006). Preparative isolation and purification of chemical constituents from the root of *Polygonum multiflorum* by high-speed counter-current chromatography. J. Chromatogr. A.

[5] Chen Y., Wang M., Rosen R.T., Ho C.T. (1999). 2,2-diphenyl-1-picrylhydrazyl radical-scavenging active components from *Polygonum multiflorum* Thunb. J. Agric. Food Chem..

[6] Chan Y.-C., Wang M.-F., Chang H.-C. (2003). *Polygonum multiflorum* Extracts improve cognitive performance in senescence accelerated mice. Am. J. Chin. Med..

[7] Chan E., Wong C.Y.-K., Wan C.-W., Kwok C.-Y., Wu J.-H., Ng K.-M., So C.-H., Au A.L.-S., Poon C.C.W., Seto S.-W. (2010). Evaluation of anti-oxidant capacity of root of *Scutellaria baicalensis* Georgi, in comparison with roots of *Polygonum multiflorum* Thunb and *Panax ginseng* CA Meyer. Am. J. Chin. Med..

[8] Lv L., Shao X., Wang L., Huang D., Ho C.-T., Sang S. (2010). tilbene glucoside from *Polygonum multiflorum* Thunb. A novel natural inhibitor of advanced glycation end product formation by trapping of methylglyoxal. J. Agric. Food Chem..

[9] Ahmed N., Thornalley P.J. (2007). Advanced glycation endproducts: What is their relevance to diabetic complications?. Diabetes Obes. Metab..

[10] Wong C.C., Li H.B., Cheng K.W., Chen F. (2006). A systematic survey of antioxidant activity of 30 Chinese medicinal plants using the ferric reducing antioxidant power assay. Food Chem..

[11] Wang H.Y., Liu T.T., Song L.X., Huang D.J. (2012). Profiles and alpha-amylase inhibition activity of proanthocyanidins in unripe *Manilkara zapota* (Chiku). J. Agric. Food Chem..

[12] Murata T., Miyase T., Yoshizaki F. (2011). Hyaluronidase inhibitory rosmarinic acid derivatives from *Meehania urticifolia*. Chem. Pharm. Bull..

[13] Ye M., Han J., Chen H., Zheng J., Guo D. (2007). Analysis of Phenolic compounds in rhubarbs using liquid chromatography coupled with electrospray ionization mass spectrometry. J. Am. Soc. Mass Spectrom..

[14] Chen W., Fu C., Qin Y., Huang D. (2009). One-pot depolymerizative extraction of proanthocyanidins from mangosteen pericarps. Food Chem..

[15] Liu T., Song L., Wang H., Huang D. (2011). A high-throughput assay for quantification of starch hydrolase inhibition based on turbidity measurement. J. Agric. Food Chem..

[16] Fu C., Loo A.E.K., Chia F.P.P., Huang D. (2007). Oligomeric proanthocyanidins from mangosteen pericarps. J. Agric. Food Chem..

[17] Fu C.L., Chen W., Quek Y.L., Ni R.Y., Ghani A.B.A., Leong W.W.Y., Zeng H.Q., Huang D.J. (2010). Sustainability from agricultural waste: Chiral ligands from oligomeric proanthocyanidins via acid-mediated depolymerization. Tetrahedron Lett..

